# Systemic Delivery of Recombinant Brain Derived Neurotrophic Factor (BDNF) in the R6/2 Mouse Model of Huntington’s Disease

**DOI:** 10.1371/journal.pone.0064037

**Published:** 2013-05-20

**Authors:** Carmela Giampà, Elena Montagna, Clemente Dato, Mariarosa A. B. Melone, Giorgio Bernardi, Francesca Romana Fusco

**Affiliations:** 1 Laboratory of Neuroanatomy, Santa Lucia Foundation IRCCS Hospital at the European Center for Brain Research, Rome, Italy; 2 Department of Neuroscience, University of Rome Tor Vergata, Rome, Italy; 3 Division of Neurology, Department of Clinical and Experimental Medicine and Surgery, Second University of Naples, Naples, Italy; 4 Institute of Protein Biochemistry, CNR, Naples, Italy; University G. D’Annunzio, Italy

## Abstract

Loss of huntingtin-mediated BDNF gene transcription has been shown to occur in HD and thus contribute to the degeneration of the striatum. Several studies have indicated that an increase in BDNF levels is associated with neuroprotection and amelioration of neurological signs in animal models of HD. In a recent study, an increase in BDNF mRNA and protein levels was recorded in mice administered recombinant BDNF peripherally. Chronic, indwelling osmotic mini-pumps containing either recombinant BDNF or saline were surgically placed in R6/2 or wild-type mice from 4 weeks of age until euthanasia. Neurological evaluation (paw clasping, rotarod performance, locomotor activity in an open field) was performed. After transcardial perfusion, histological and immunohistochemical studies were performed. We found that BDNF- treated R6/2 mice survived longer and displayed less severe signs of neurological dysfunction than the vehicle treated ones. Primary outcome measures such as brain volume, striatal atrophy, size and morphology of striatal neurons, neuronal intranuclear inclusions and microglial reaction confirmed a neuroprotective effect of the compound. BDNF was effective in increasing significantly the levels of activated CREB and of BDNF the striatal spiny neurons. Moreover, systemically administered BDNF increased the synthesis of BDNF as demonstrated by RT-PCR, and this might account for the beneficial effects observed in this model.

## Introduction

Huntington’s disease (HD) is an inherited neurodegenerative disorder characterized by motor dysfunction, cognitive decline and emotional and psychiatric disorder [1]. The striatum is the major site of HD degeneration [2], [3], where projection neurons massively die [4].

The mutation involves the IT15 gene [5] encoding for the protein huntingtin, and is characterized by a CAG expansion beyond the normal 10–35 repeat range [4]. HD pathology is characterized by the formation of intranuclear inclusions of mutated huntingtin in the brain [6]. Such inclusions have been shown to interact with and impair the function of a number of transcription factors [7].

Striatum is a brain region which is highly susceptible to neurodegenerative processes. Striatal neurons are prone to undergo cell death induced by acute brain insults such as ischemia and hypoglycemia [8], [9]. Interestingly, HD is histopathologically characterized by marked loss of the striatal projection neurons in a way that is similar to that of ischemia [10].

Brain-derived neurotrophic factor (BDNF) plays a major role in the survival of mature neurons in the central nervous system, and in the striatum, in particular [11].

Striatal projection neurons are particularly vulnerable to neurodegeneration induced by HD. One of the mechanisms underlying such vulnerability is explained by the fact that these neurons do not synthetize sufficient amounts of BDNF, and that striatal BDNF depends on the cortical synthesis and release [12], [13]. BDNF is synthesized by cortical neurons and released in the striatum by cortico-striatal anterograde transport [12], [13], as very low levels of BDNF mRNA have been demonstrated in the adult rat striatum [14]. For this reason, cortical pyramidal neurons function is necessary for an appropriate BDNF supply for the striatum [11].

Interestingly, huntingtin directly modulates the expression of neuron-restrictive silencer factor (NRSF)-controlled neuronal genes, including BDNF gene [15]. Thus, wild-type huntingtin stimulates production of BDNF [16], whereas mutant huntingtin decreases it [17]. In fact, BDNF is decreased in brain tissue from human HD patients [17], [18] and in some mice transgenic for mutant huntingtin [17], [19], [20]. Overexpression of BDNF proved protective in the R6/1 mouse model of HD [21].

Indeed, a specific involvement of BDNF was demonstrated in the pathophysiology of the disease: a loss of huntingtin- mediated BDNF gene transcription has been observed both in a mouse model of HD and in HD patients [17]. Moreover, Canals and coworkers [22] showed that BDNF knockout mice display an earlier age of onset and more severe motor symptoms. Conversely, BDNF proved to be neuroprotective in several disease models [23], [24].

This enlarged neurological pathology correlates with morphological alterations, supporting the evidence that BDNF plays a role in the specific degeneration of the striatal projection neurons.

Lower levels of serum Brain Derived Neurotrophic Factor (BDNF) were described in HD patients compared to controls were reported [25]. In that study, the severity of clinical symptoms correlated negatively with the levels of BDNF.

BDNF represents a powerful neuroprotective compound not only in HD, but also in other conditions such as brain ischemia [26], [27], [28] traumatic spine injury [29], depression [30].

BDNF ability to cross the blood brain barrier has been debated. Indeed, transport of BDNF across the brain capillary endothelial wall, which forms the blood-brain barrier (BBB) in vivo, is negligible for some authors [31]. However, other authors [32] reported that BDNF is able to cross the BBB.

Interestingly, in a recent study, an increase in BDNF mRNA and protein levels was recorded in the brain of mice administered recombinant BDNF peripherally [30]. These results were very encouraging, as the possibility of increasing brain BDNF by a systemic administration would be a powerful tool to fight neurodegeneration in HD.

In this paper, we explored such possibility by administering recombinant BDNF to a transgenic mouse model of HD through systemic delivery.

## Results

### Behavioral Studies

**Figure 1:**
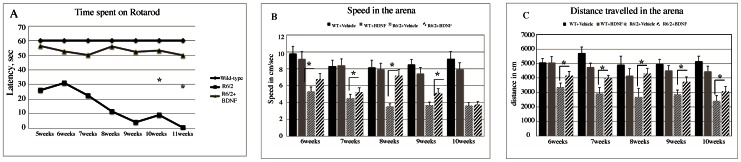
Effects of BDNF treatment on motor behavior. In (A) Latency to fall from the accelerating rotarod in the R6/2 mice. A two way ANOVA indicates an overall significant effect of treatment. R6/2 mice treated with vehicle exhibited a progressive decrease in the latency to fall and this decrease was ameliorated by BDNF treatment. (B–C) Histograms represent mean values +SEM for (B) distance traveled and (C) speed in in the open field during a 10 min test session. Wild type mice treated with vehicle exhibited only a slight decrease in distance traveled and speed over the repeated exposures to the open field. In contrast R6/2 mice treated with vehicle exhibited a progressive decrease both in distance traveled and speed. This difference between wild type and R6/2 mice was largely ameliorated by BDNF treatment.

Motor coordination was assessed as the ability of mice to maintain balance on an accelerating rotarod at 6 through 13 weeks of age. Figure 1 A shows the rotarod performance in R6/2 and wild-type mice treated with BDNF or saline. A two-way anova with genotype and treatment as main factors revealed that R6/2 mice had a significant impairment in motor coordination compared with wild-type mice and that BDNF affected performance in a genotype-dependent fashion (significant genotype · treatment interaction: F_1,22_ = 62.2, P<0.000). Post hoc pair comparisons then indicated no difference in performance between R6/2 mice treated with BDNF and wild- type mice treated with saline or BDNF suggesting a recovery of motor coordination in the BDNF treated mutants. Accordingly, performance in these groups was significantly higher than in R6/2 mice treated with saline (P<0.01 for each comparison). Motor activity data collected in the open field task and including the total distance traveled, speed of locomotion in the arena are shown in Fig. 1 B–C. Statistical results indicated that R6/2 mice traveled a shorter distance and at a lower speed than wild-type mice, confirming our previous data [3], and that BDNF restored performance in a genotype-dependent fashion (significant genotype · treatment interaction, distance traveled: F_2,16_ = 180,82, P<0.000; speed: F_2,14_ = 17.57, P<0.000). In fact, pair comparisons revealed that R6/2 mice treated with BDNF did not differ from wild-type mice treated with saline for any index (distance or speed) of motor performance (P<0.00 for all comparisons), signifying that the treatment enabled recovery of motor activity (Figure 1 B and C).

### Immunohistochemistry and Western Blotting Studies

#### Analysis of CREB activation in the surviving striatal spiny neurons and in the cortical neurons

**Figure 2:**
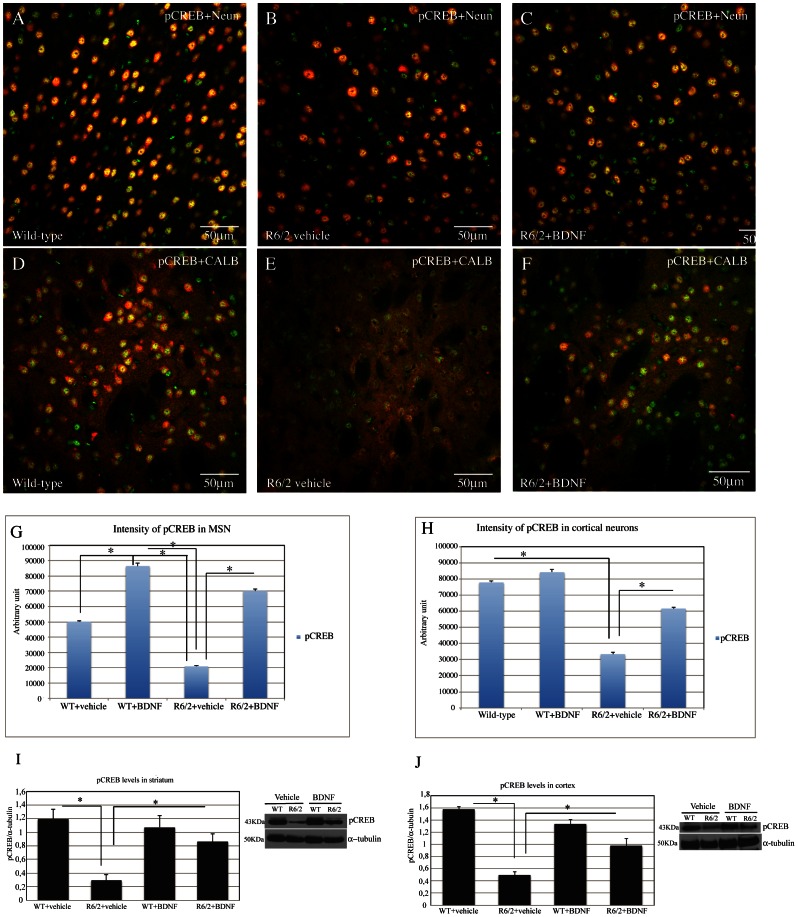
Effects of BDNF treatment on pCREB in the cortex and striatum of R6/2 mice. Representative confocal laser scanning microscopy images of dual-label immunofluorescence for Neun (red) and pCREB (green) or CALB (red) and pCREB in the cortex (A–C) or striatum (D–F) of wild-type, (A–D) vehicle R6/2 mice (B–E), or (C–F) R6/2 mice treated with BDNF at 13 weeks of age. (G–H) Quantification of the intensity of pCREB immunoreactivity associated with Neun-labeled cortical neurons (G) or CALB-labeled striatal neurons. (I–J) pCREB levels were analyzed by Western blotting of protein extracts obtained from the striatum and cortex of WT and R6/2 mice treated with vehicle or BDNF at 12 weeks of age.

As shown in figure 2, our immunohistochemical double labeling study revealed that the intensity of pCREB, expressed in arbitrary units, in the surviving spiny neurons was significantly decreased in the saline treated R6/2 compared to the wild type littermates, and that pCREB levels were significantly higher in systemic BDNF treated R6/2 and also in BDNF treated wild type mice compared to both the saline R6/2 and wild-type animals with a treatment effect F_1,867_ = 172.39 P<0.000 (Fig. 2 D, E, F and G). Moreover, we observed increased levels of activated CREB not only in the striatal projection neurons, but also in cortical neurons of systemic BDNF treated R6/2 mice (Figure 2 A–B–C and H). Interestingly, pCREB intensity in systemic BDNF treated R6/2 mice was significantly higher compared both to the saline- treated R6/2 and to the saline-treated wild type mice. The results were confirmed by western blotting (fig. 2 I–J).

#### Analysis of phosphorylated ERK in the striatum

**Figure 3:**
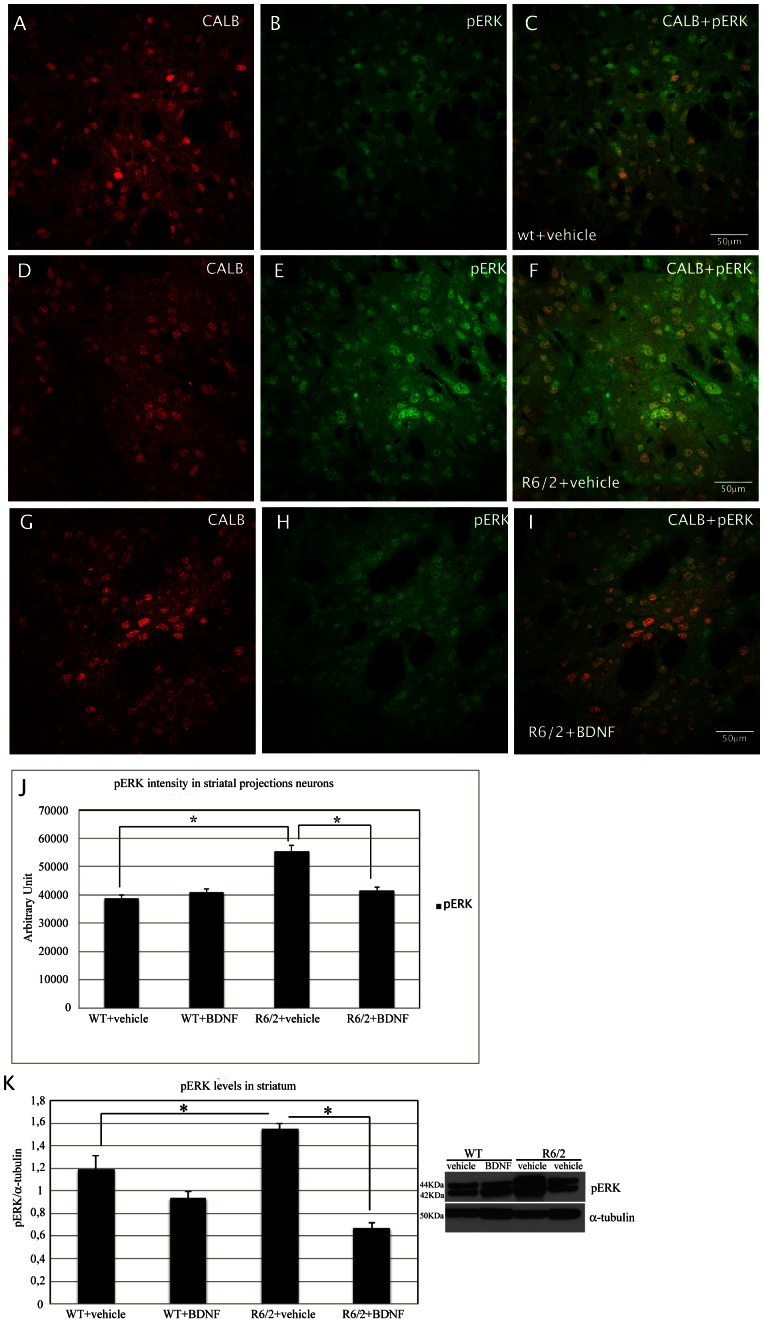
Effects of BDNF treatment on pERK in the striatum of R6/2 mice. Representative confocal laser scanning microscopy images of dual-label immunofluorescence for CALB (red) and pERK (green) in striatum of wild-type, (A–C) vehicle R6/2 mice (D–F), or (G–I) R6/2 mice treated with BDNF at 13 weeks of age. (J) The histogram describes the levels of pERK (expressed in arbitrary units) in projection neurons of the wild-type mice, R6/2 HD mice treated with saline and R6/2 HD mice treated with BDNF respectively. There is a higher density of immunoreactivity in the R6/2 vehicle treated samples relative to the wild type samples. This increment is largely eliminated in samples from R6/2 mice treated with BDNF. pERK levels were analyzed by Western blotting of protein extracts obtained from the striatum and cortex of WT and R6/2 mice treated with vehicle or BDNF at 12 weeks of age.

Changes in pERK paralleled the ones observed in CREB. Indeed, as shown in figure 3, our immunohistochemical double labeling study revealed that the intensity of pERK in the surviving spiny neurons was significantly increased in the saline treated R6/2 compared to the wild type littermates, and that pERK levels were significantly lower in systemic BDNF treated R6/2 and also in BDNF treated wild type mice compared to both the saline R6/2 and wild-type animals, with a treatment effect F_1,623_ = 13.96 P<0.000 (Fig. 3). These results are consistent with our previous observations of reduced levels of pERK in the R6/2 mouse model after treatment with other neuroprotective agents [33] The results were confirmed by western blotting (Fig. 3 K).

#### Levels of BDNF

**Figure 4:**
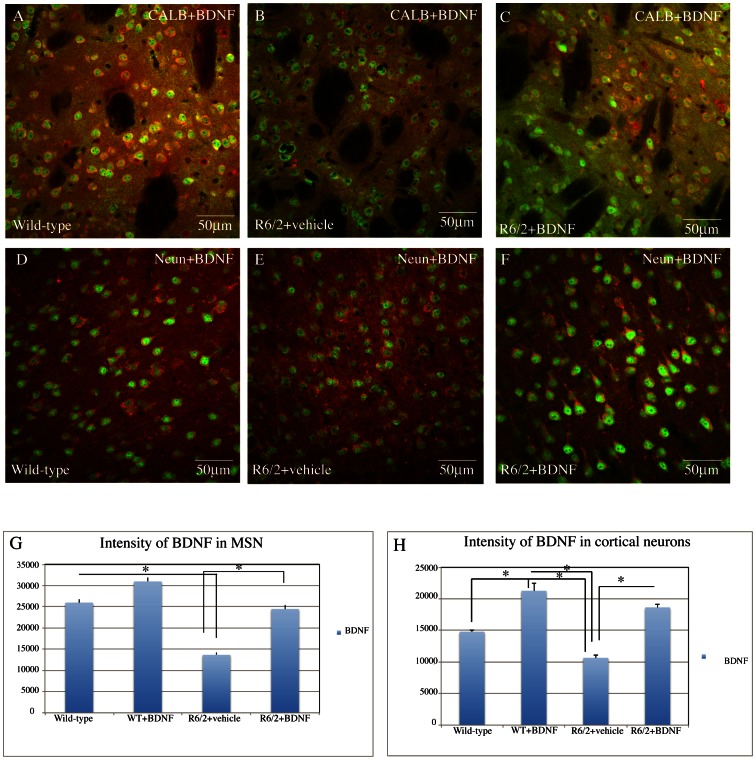
Effects of BDNF treatment on BDNF levels in the cortex and striatum of R6/2 mice. Representative confocal laser scanning microscopy images of dual-label immunofluorescence for Neun (red) and BDNF (green) or CALB (red) and BDNF in the cortex (D–F) or striatum (A–C) of wild-type, (A–D) vehicle R6/2 mice (B–E), or (C–F) R6/2 mice treated with BDNF at 13 weeks of age. (G–H) Quantification of the intensity of BDNF immunoreactivity associated with Neun-labeled cortical neurons (H) or CALB-labeled striatal neurons (G). There is a reduced density of immunoreactivity in the R6/2 vehicle treated samples relative to the wild type samples. This decrease is largely eliminated in samples from R6/2 mice treated with BDNF.

**Figure 5:**
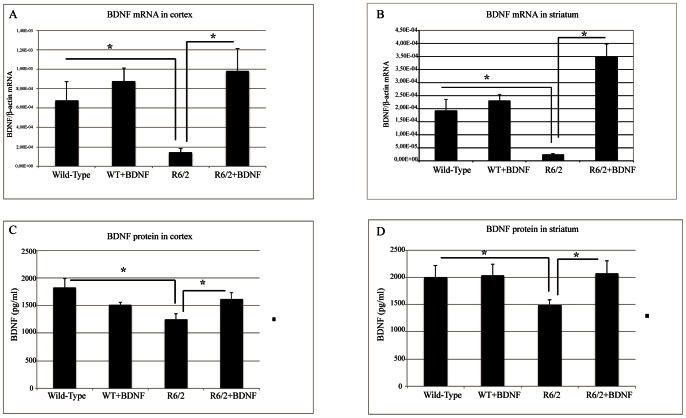
Quantitative RT-PCR analyisis of expression of mouse BDNF transcripts in wild-type and R6/2 mice treated with vehicle or BDNF (A–B). Copy numbers of mature BDNF transcript were normalized by dividing the copy number of the reference gene β-actin. (C–D) BDNF protein levels determined by enzyme-linked immunosorbent assay (ELISA). Statistical analysis revealed an increasing expression of protein levels in cortex (C) and striatum (D) of R6/2 mice treated with recombinant BDNF.

We aimed at investigating whether the treatment with systemic BDNF affected BDNF expression in the brain. As shown in figure 4, systemic BDNF proved effective in augmenting the protein expression of BDNF significantly in the treated group compared to the saline R6/2 mice. In fact, there was no statistical difference between vehicle treated wild type mice and systemic BDNF treated R6/2, indicating that systemic BDNF restored BDNF levels to normal values. Treatment effect in the cortex was F_1,1064_ = 157.97 P<0.000 (fig. 4 H), in the striatum F_1,897_ = 1815.66 P<0.000 (fig. 4 G). The results were also confirmed by ELISA protein assays (fig. 5, C–D).

### Expression Analysis of BDNF mRNA

We have evaluated the levels of the BDNF mRNA in cerebral cortex and striatum from wild-type and R6/2 mice treated with vehicle or recombinant BDNF. We aimed at determining whether the steady-state levels of the transcripts increased after treatment with BDNF in symptomatic R6/2 mice. As shown in Fig. 5 A, there was a significant increase in BDNF transcript in the cortex of R6/2 mice treated with exogenous BDNF compared to that observed in vehicle treated R6/2 mice (F(1,60) = 4,92;P<0,05). Interestingly, we observed that there was an increase of BDNF transcripts in R6/2 mice treated with recombinant BDNF even in the striatum, that normally expresses small amounts of mRNA for BDNF, (F(1,73) = 13,07; P<0,000) (Fig. 5 B).

### Neuropathological Outcome Measures

#### Striatal volume

**Figure 6:**
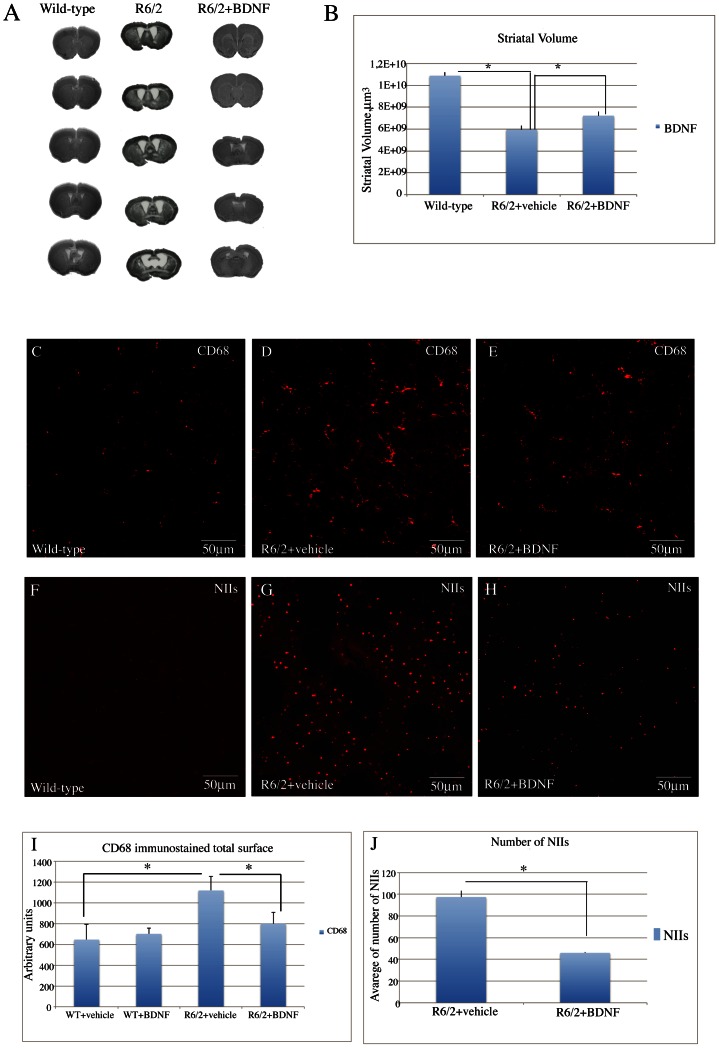
Effects of recombinant BDNF treatment on striatal atrophy in R6/2 mice. Transmitted light microscope images showing representative Nissl-stained coronal sections of a wild-type mouse, a vehicle treated R6/2 mouse and a BDNF-treated R6/2 mouse, (A), left to right, respectively. Marked gross atrophy and enlarged lateral ventricles are present in the sections from the vehicle treated R6/2 mouse compared the wild type mouse. These differences are largely absence from the sections of the R6/2 mouse treated with BDNF. (B) Quantification of differences in striatal volume from wild type and vehicle- or BDNF-treated R6/2 mice. Post hoc analysis indicated that R6/2 mice treated with vehicle had a significantly reduced striatal volume compared to the wild type group. The striatal volume of R6/2 mice treated with BDNF was significantly greater than that of the vehicle treated R6/2. *Effects of BDNF treatment on reactive microglia in the striatum of R6/2 mice (C–E).* Representative photomicrographs of single label immunofluorescence for the marker of activated microglia, CD68, in the striatum of (C) wild type, (D) vehicle treated R6/2, or (E) R6/2 mice treated with BDNF. In the sample from the vehicle treated R6/2 mouse, an intense microglial reaction is observed, while in the sample from a BDNF treated R6/2 mouse there were fewer reactive cells, along with quiescent cells. *Effects of BDNF treatment on the density of NIIs in the striatum of R6/2 mice. (*F–H). Confocal laser scanning microscopy images of single-label immunofluorescence for NII marker (EM48). Single-label immunostaining was employed in the striata of vehicle-treated wild-type (WT) mice (F), vehicle-treated R6/2 mice (G) and BNF-treated R6/2 mice (4 µg/day) (H) at 13 weeks of age. Of note, is the lower density of EM48-positive NIIs in the BDNF-treated R6/2 mice (H). (I) Quantification of NIIs in vehicle- or BDNF treated R6/2 mice. There were no NIIs detected in striatum of wild type mice, so this group was not included in the statistical analysis. One Way Anova indicated that the density of NIIs in striatum of R6/2 mice treated with BDNF was lower than that in R6/2 mice treated with vehicle (p<0.0001). (J) Histogram showing the quantification of CD68 immunostained total surface in vehicle or BDNF treated wild-type and vehicle or BDNF treated R6/2 mice. Data are presented as the mean values of CD68 positive areas ± SEM. Microglia reactivity was significantly decreased in the striatum of R6/2 mice after treatment with BDNF (*P<0.001).

As shown in figure 6 (A–B) the mean striatal volume of saline treated R6/2 was significantly smaller than that of wild type mice. The striatal volume of systemic BDNF treated R6/2 mice was restored to levels comparable of wild type mice. Treatment effect was F_2,6_ = 7.14 P<0.05.

### Study of the Microglial Reaction

CD-68 immunostaining was performed for the detection of microglia in our samples. (Figure 6C). Our immunostaining revealed an intense microglial reaction in the saline treated R6/2 group, where microglial cells appeared numerous, with coarse arborizations and a rod-shaped body (Figure 6 D). The microglial reaction was attenuated in systemic BDNF treated R6/2 mice with few reactive cells and arborizations along with normal quiescent cells (figure 6 E).

#### Study of NIIs in the striatum

We examined whether systemic BDNF treatment affected NIIs formation in the striatum. No NIIs -like ubiquitin immunoreactivity was found in the wild type animals (figure 6 F). R6/2 animals treated with saline showed a much higher density of NIIS in the striatum compared to BDNF-treated mice (figure 6 G). From the analysis of ubiquitin immunohistochemistry in sections counterstained with Nissl (not shown) we observed that the percentage of NIIs in the striata of R6/2 mice treated with saline was significantly higher compared to the systemic BDNF treated group (figure 6 J). Treatment effect was F_1,_35 = 53.08 P<0.000.

## Discussion

The present results show that systemic BDNF is neuroprotective in the R6/2 mouse model of HD.

Our study confirms and extends previous observations about the protective role of BDNF. Indeed, both BDNF delivered intrastriatally and selective overexpression of BDNF are able to reverse striatal damage and improve motor performance in transgenic HD mice [21], [22], [34].

Our group had previously demonstrated how increased levels of BDNF, through the use of phosphodiesterase inhibitors, exerts beneficial effects in the rat quinolinic acid model and in the R6/2 mice HD model [35], [36], [37], [38].

With this in mind, we aimed at investigating if the direct administration of BDNF would exert a beneficial effect in the R6/2 mouse model of HD.

In the present study, we found that recombinant BDNF had a significant effect on the development of neurological impairment in the R6/2 mouse. Indeed, BDNF reduced deficits in rotarod performance and in open field activity.

Corresponding to these in-life effects, BDNF treatment ameliorated neuropathology in the R6/2 mice. The treatment sensibly ameliorated the loss of striatal area and nearly completely restored the loss of and morphological changes in the medium spiny neurons, including the quantitative reduction in soma size.

Striatal neurons in the R6/2 mice accumulate NIIs, which are aggregates of the polyglutamine peptide encoded by the huntingtin exon 1 transgene. BDNF treatment significantly reduced the density of these aggregates in striatum.

Moreover, this treatment reduced significantly the microglial reaction that accompanies neuronal death in HD. Microglia are the resident immune cells of the CNS. They resemble peripheral tissue macrophages and are the primary mediators of neuroinflammation [39], [40]. The involvement of neuroinflammation in HD, although less studied than in other neurodegenerative diseases, is gaining momentum [41], [42]. Microglial calcium, accumulation of ferritin and complement are all possible mechanisms underlying the involvement of microglia in HD [43]. Therefore, the reduction of microglial reaction observed in our study not only confirms the beneficial effect of BDNF treatment, but also contributes to explain the involvement of microglia in HD.

The present data show that peripheral BDNF administration significantly increased levels of BDNF in the striatum and cortex, and increased levels of phosphorylated CREB, which is target of BDNF-TrkB signaling [44], [45].

Moreover, BDNF induced a downregulation of ERK phosphorylation in the spiny projection neurons of R6/2 mouse.

Neuronal degeneration does not afflict all types of striatal neurons in the same fashion. In fact, while GABAergic spiny projection neurons degenerate massively interneurons display different levels of resistance to HD. In a previous paper, we observed that projection neurons as well as parvalbuminergic interneurons, which are most vulnerable to HD de- generation, contain pERK levels that tend to increase with age (in the wild-type animals) and with the progression of the disease (in the R6/2 mice). Such increase was reversed by PDE inhibition [33].

Thus, our present study confirms that the neuroprotective effects of increased BDNF translate into a downregulation of pERK.

A significant increase of pCREB in systemic BDNF treated animals was observed in the cortex. Such observation strongly suggests that systemic BDNF-modulated pCREB increase [46], [47], [30] in the cortex leads to higher levels of BDNF which is transported to the striatum and locally exerts its beneficial effects for the striatal cells.

The effect of increasing levels of pCREB is consistent with the possibility that peripheral BDNF enters the brain and directly activates TrkB–ERK–CREB signaling.

The question whether systemic BDNF is capable to cross the blood-brain barrier (BBB) has been the subject of several studies [33], [48]. In particular, many authors deny the existence of BDNF transport into brain [49], [48]. Other authors [50] showed that BDNF does enter the brain. Indeed, Givalois and coworkers [51] have shown that recombinant BDNF administered through Alzet™ micropumps was able to modulate the expression of brain peptides in the hypothalamus in a more efficient way than the acute intracerebroventricular injections. Thus, even though the question remains open, there are several lines of evidence that peripherally administered BDNF exerts its effects by entering the BBB.

Indeed, if BDNF does not penetrate the BBB, it is also possible that peripheral BDNF acts indirectly by increasing the expression of cortical and striatal BDNF. Moreover, it could be possible that peripherally administered BDNF increases the expression of other growth factors, which also activate CREB, which in turn would stimulate BDNF in the cortex and in the striatum.

Moreover, we demonstrate that, following BDNF administration, BDNF mRNA is increased in the brain, as showed by our RT PCR data. This observation is very important as it suggests the existence of a positive feedback triggered by systemic BDNF. The increase in BDNF mRNA was observed not only in the cortex, from which most of the BDNF necessary for neuronal survival is produced, but also in the striatum, which typically does not contain large amounts of BDNF transcripts.

The mechanism underlying these effects remain unclear and deserves further investigation.

The bulk of these data showed that systemic BDNF does have, indeed, a pronounced neuroprotective effect on R6/2 mice. Neuroprotective effects of systemic BDNF have been described in several models of neurological disorders [29], [30].

Thus, our data are not only consistent with these reports, but they add a closer, specific perspective to the potential use of systemic BDNF to fight HD degeneration.

## Methods

### Animals and Drug Administration

Heterozygous transgenic R6/2 [52] males of CBAXC57BL/6 strain were and bred with CBAXC57BL/6 F1 females, all obtained from Jackson Laboratories (Bar Harbor, ME). All the experiments were conducted on F1 mice, to limit the possibility of CAG number and phenotype variations. The offspring were genotyped by PCR assay of DNA obtained from tail tissue. Mice were weaned and, after genotyping, treatments began at 4 weeks of age. The study groups included: R6/2 mice that were administered saline, R6/2 mice treated with BDNF, BDNF treated wild type mice and saline-treated wild-type mice. Recombinant BDNF (Regeneron Pharmaceuticals) was diluted in order to have a dosage of 4.0 µg per 24 h (152 µg in 100 µl per micropump) in phosphate- buffered saline with 0.1% bovine serum albumin (protease- free, Sigma Aldrich, St Louis, MO).

For the surgery, mice were anesthetized with xylazine and zolazepam+tyletamine. An incision in the skin was made between the scapulae, and a chronic, indwelling osmotic mini-pump (Alzet Model 1004; Durect, Cupertino, CA) containing either recombinant BDNF or saline was implanted subcutaneously. The incision was closed using nylon sutures. The osmotic mini-pump administered recombinant BDNF or saline subcutaneously over a period of 28 days. At the end of this period, the micropumps were replaced by new ones, following the procedure described above.

Mice were handled under the same conditions by one investigator at the same time of the day. Mice were identified by a randomly assigned code so that the studies were performed blind as to the genetic identity. Mice were housed five in each cage under standard conditions with ad libitum access to food and water. All the behavioral and histological data were collected by observers who were blinded to treatment.

### Survival

Because micropumps had to be replaced every 28 days, a study of the survival could not be performed, as the procedure involving such replacement was too stressful for the animals at the terminal stages of the disease. Thus, we only replaced micropumps once in the lifetime of our mice and sacrificed them all at the same time point of 13 weeks.

### Behavioral Studies

For the behavioral studies, 25 animals (9 R6/2 treated with BDNF, 7 R6/2 treated with saline, 5 wild type treated with BDNF, 4 wild type treated with saline).

1. Motor coordination and balance were estimated using a five-station mouse rotarod (Rotarod/RS LSI Letica, Biological Instruments, Varese, Italy). In each station, the rod was 6 cm in length and 3 cm in diameter. Mice were first trained at increasing speed up to a constant speed of 16 rpm for three consecutive trials when they were 7 weeks old. Subsequently, they were administered one rotarod trial twice a week from 8 to 11 weeks of age. The test session was delivered when mice were 12 weeks old and fully symptomatic. In this session, the speed of rotation was increased from 4–25 rpm over 60 s. Mice were given three trials on the rod, and their latencies to fall were measured and averaged. For the mice that did not fall, a maximum latency of 60 s was defined, at which the individual test terminated.
2. Motor activity was measured in an open field consisting of a circular arena (60 cm diameter) with the floor painted white and divided into central and peripheral sectors by drawing black lines. The total area of the two sectors was similar (approximately 1400 cm^2^). The apparatus was placed in a soundproof room illuminated by a red ceiling light (80 W). A video camera above the arena was connected to a video recorder and to a monitor located in an adjacent room. Mice were placed in the arena for 5 mins during which the distance traveled, speed and time spent in the central sector was recorded by means of dedicated software (Noldus, Wageningen, the Netherlands).

### Quantification of Total BDNF mRNA Levels

Total RNA was extracted from striatum and cortex of 16 animals (4 animals for each group: wild type treated with vehicle or BDNF and R6/2 treated with vehicle or BDNF) using the Trizol reagent (Invitrigen, Burlington, Ontario, Canada) following the manufacturer’s protocol. RNA concentration was evaluated by optical density at 260 nm and the purity was determined by 260 nm/280 nm of absorbance, the total RNA was stored at −80°C.

Total RNA (1 µg) was reverse-transcribed into single-stranded cDNA using Superscript III reverse transcriptase (Invitrogen, CA, USA). Briefly, 1 ul oligo-d(T)_18–20_ primers, 1 µl of dNTP(10 mM), 15 µl of RNA and water until 13 ml was incubated at 65°C for 5 min. The solution was briefly chilled on ice, then added to 4 µl first strand synthesis buffer, 1 ul 0,1 M DTT, 1 µl RNAaseOUT and 1 ul SuperScript III RT (200 U/ml). The reaction was incubated at 50°C for 60 min, followed by 70°C for 15 min to terminate the reaction. Parallel reactions for each RNA sample were run in the absence of SuperScript III to assess the degree of contaminating genomic DNA. The cDNA products were used as templates for PCR amplification.

Quantitative reverse transcription-PCR (qRT-PCR) was used to determine the number of copies of mature BDNF transcript in cDNA samples derived from the striatum and cortex of wild-type and HD transgenic mice using the LightCycler thermal cycler system (Roche Applied Science, Indianapolis, IN, USA). Gene-specific PCR primers were designed based on the sequence of mouse BDNF mRNA available in the GenBank database. The primers pair (forward/reverse) used to amplify BDNF are: BDNF forward GATGAGGACCAGAAGGTTGG; BDNF reverse GATTGGGTAGTTGGGCATTG. A no-template control with water was performed parallel in all experiments. A no-template control with water was performed parallel in all experiments.

All of the reactions were performed in a total volume of 20 ul containing 2 µl of cDNA, 1 ul of forward and reverse primers and 1 ml of each RT product.

The amplification cycles consisted of an initial denaturing cycle at 95°C for 10 min, followed by 45 cycles of 15 s 94°C, 15 s at 56°C and 20 s at 72°C. Fluorescence was quantified at the end of each cycle and product formation was confirmed by means of melting curve analysis (65°C–99°C). Copy numbers of mature BDNF transcript were normalized by dividing the copy number of the reference gene beta-actin (forward: AGTGTGAGGTTGACATCGGTA; reverse: GCCAGAGCAGTAATCTCCTTCT).

### BDNF Protein ELISA Assay

Lysates from frozen tissues were prepared in lysis buffer consisting of Triton-X100 1%; Tris±HCl, 50 mM, pH 7.5; NaCl, 300 mM; glycerol 5%; EDTA, 5 mM; EGTA, 1 mM; supplemented with phenylmethane-sulphonyl fuoride (PMSF)1 mM; protease inhibitor cocktail, 1∶ 100 (Sigma), NaF(Na-fluoride) 10 mM, Na-orthovanadate 1 mM; Na-Deoxycholate 0,5%; β-glycerophosphate 1 mM; SDS 0,1%. For each µg of tissue sample 10 µL of lysis buffer was used. Samples were homogenized and sonicated four times at 50%–60% output and 60% duty cycle. After sonication the samples were acidified (pH 3,0), neutralized (pH 7,5) and centrifuged for 20 minutes at 4°C at maximum Biofuge speed. The supernatants were collected and protein concentration evaluated using the Pierce BCA protein Assay KIT (Thermo Scientific, USA). Samples were stored at-80C.

Samples were assayed for BDNF content with the mouse BDNF Elisa immunoassay Kit (Immunological Science, Italy). Assays were performed as described by the manufacturer.

### Western Blotting

Tissue lysates were prepared as mentioned above. Equal amounts of protein (60 µg) were separated by 10% SDS- PAGE gels and transferred to PVDF membranes (Millipore, Billerica, USA). Nonspecific sites were blocked by 0.1% Tween 20 and 5% milk powder in TBS for 2 h at room temperature. Membranes were then incubated overnight at 4°C with the specific anti-pERK rabbit anti- body (1∶1000, Immunological Science, Italy); rabbit anti-pCREB (Millipore, USA) in TBS/1% Tween-20 with 1% of milk powder. After primary antibody incubation, the membranes were incubated for 1 h at room temperature with the appropriate horseradish peroxidase-conjugated secondary antibody (1∶5000, Immunological Science) and the reaction was visualized by using an enhanced chemiluminescent detection system (Millipore, Bilerica, MA). Incubation with a mouse anti α-tubulin (1∶1000; Cell Signaling, Beverly, MA) was performed to obtain loading controls. Densitometric quantification of the immunoblots was performed using Image J software.

### Histological and Immunohistochemical Studies

#### Tissue processing

For the histological examination, 25 animals (9 R6/2 treated with BDNF, 7 R6/2 treated with saline, 5 wild type treated with BDNF, 4 wild type treated with saline) 12–14 weeks of age were transcardially perfused under deep anesthesia with saline solution containing 0.01 ml heparin, followed by 60 ml of 4% paraformaldehyde in saline solution. The brains were removed and postfixed overnight at +4°C, cryoprotected in 10% sucrose and 20% glycerol in 0.1 M phosphate buffer (PB) with sodium azide 0.02% for 48 h at 4 8C. Brains were sectioned frozen on a sliding microtome at 40 µm thickness to obtain serial sections.

For the immunohistochemical studies, primary omission controls, normal mouse and rabbit serum controls and preimmune serum controls were used to confirm the specificity of our immunohistochemical labeling.

#### 1. Analysis of CREB activation in the surviving striatal spiny neurons

Double label immunofluorescence was employed to identify the intensity of activated CREB in the striatal spiny projection neurons [53]. Briefly, sections were incubated with a cocktail of anti calbindin-28 kDa antibody (mouse anti-CALB, SIGMA, St. Louis, MO) and an antibody against Ser-133 phosphorylated CREB (rabbit anti-Phospho-CREB, Upstate, NY) both at a 1∶200 concentration in a 0.1 M phosphate buffered (PB) solution containing Triton X 0.3% and 0.02 sodium azide for 72 h at +4°C. After 3 15-min rinses in PB, sections were incubated with a cocktail of goat anti-rabbit Cy2-conjugated secondary antibody and donkey anti-mouse Cy3-conjugated secondary antibody (both Jackson Immunoresearch, West Grove, PA, USA) for 2 h at room temperature.

Tissue was mounted on gelatin-coated slides, coverslipped with GEL-MOUNT TM and examined under an epi-illumination fluorescence microscope (Zeiss Axioskop 2), and a CLSM (Zeiss LSM 510) was used to acquire all the images. The intensity of pCREB staining was calculated in each of three 1.0-mm-square confocal microscope fields, in each of three rostrocaudally spaced sections on each hemisphere of 6 mice from each saline, systemic BDNF treated R6/2 mice and wild type littermates.

#### 2. Analysis of CREB activation in cortical neurons

Dual label immunofluorescence was employed to identify the intensity of activated CREB in the NeuN-labeled cortical neurons according to the previously described procedure [54].

#### 3. Analysis of Brain Derived Neurotrophic Factor (BDNF) in the striatal spiny neurons

Levels of BDNF in the projection neurons after systemic BDNF administration were measured. Double label immunofluorescence was employed using an antibody against BDNF (anti-mouse BDNF, Immunological Sciences, Italy) and CALB antibody (mouse anti-CALB, SIGMA, St. Louis, MO) using a previously described immunohistochemical protocol [55].

To evaluate the intensity BDNF immunolabeling, an image analysis of BDNF immunoreactive projection neurons was performed by means of Zeiss LSM software. The intensity of fluorescent BDNF immunolabeling in CALB positive projection striatal neurons was calculated in each of three 1.0-mm-square confocal microscope fields, in each of three rostrocaudally spaced sections on each hemisphere of 6 mice from each saline, systemic BDNF treated R6/2 mice and wild type littermates. The intensity of BDNF immunoreactivity per field, expressed in arbitrary units, was calculated by Zeiss LSM software and a mean value was obtained. BDNF immunoreactivity that was not contained in CALB immunoreactive (i.e., interneurons) neurons was calculated.

#### 4. Analysis of Brain Derived Neurotrophic Factor (BDNF) in cortical neurons

Dual label immunofluorescence was employed to identify the intensity of BDNF in the NeuN-labeled cortical neurons according to the previously described procedure.

#### 5. Analysis of pERK in the surviving striatal spiny neurons

Double label immunofluorescence was employed to identify the intensity of phosphorylated (activated) ERK in the striatal spiny projection neurons (4). Briefly, sections were incubated with a cocktail of anti calbindin-28 kDa antibody (mouse anti-CALB, SIGMA, St. Louis, MO) and an antibody against Ser-133 phosphorylated ERK (rabbit anti-Phospho-ERK, Millipore ) both at a 1∶200 concentration in a 0.1 M phosphate buffered (PB) solution containing Triton X 0.3% and 0.02 sodium azide for 72 h at +4°C. After 3 15-min rinses in PB, sections were incubated with a cocktail of goat anti-rabbit Cy2-conjugated secondary antibody and donkey anti-mouse Cy3-conjugated secondary antibody (both Jackson Immunoresearch, West Grove, PA, USA) for 2 h at room temperature.

Tissue was mounted on gelatin-coated slides, coverslipped with GEL-MOUNT TM and examined under an epi-illumination fluorescence microscope (Zeiss Axioskop 2), and a CLSM (Zeiss LSM 510) was used to acquire all the images. The intensity of pERK staining was calculated in each of three 1.0-mm-square confocal microscope fields, in each of three rostrocaudally spaced sections on each hemisphere of 6 mice from each saline, systemic BDNF treated R6/2 mice and wild type littermates.

### Neuropathological (Primary) Outcome Measures

#### 1. Evaluation of striatal volume

Standard Nissl staining was employed on coronal step serial sections from rostral neostriatum through the level of anterior commissure (interaural 4.66 mm/bregma 0.86 mm to interaural 3.34 mm/bregma -0.46 mm) from 4 animals per group. Striatal volume was measured using Neurolucida Stereo Investigator software (Microbrightfield, Cochester, VT, USA).

#### 2. Microglial reaction

The microglia was studied by immunolabeling our tissue with an antibody for microglia (rat anti-mouse CDC68 from AbD Serotec). Single label immunofluorescence was employed as previously described [56]. Tissue was mounted on gelatin-coated slides, cover-slipped with GEL-MOUNT™ and examined under an epi-illumination fluorescence microscope (Zeiss Axioskop 2). A CLSM (Zeiss LSM 700) was used to acquire all the images. *Quantification of microglia staining and statistical analysis.* Images were acquired by using a CLSM (Zeiss LSM 700) laser-scanning confocal microscope under nonsaturating exposure conditions and using the same acquisition settings for all samples. The conditions in term of gain and laser power were selected at levels that allowed optimal visualization of the fluorophore used as secondary antibody and standardized using sections from HC mice. Each image was saved at a resolution of 1024×1024 pixels. These settings were then applied as standards for all subsequent images. By a 40X objective Z-stacks images of striatum from coronal sections were collected using computer-controlled microstepper stage of the confocal microscope. Stacks of images were, then, combined into a single two-dimensional (2D) projection image, exported in TIF file format using NIH ImageJ software and used to quantify the area of histochemically positive tissue for CD68.

The area of immunolabeling was calculated in each of three separate fields (one dorsolateral, one central and one medial, each 1 mm in diameter) on each hemisphere in each of three rostrocaudally spaced sections from 4 mice from each experimental group (wild-type, wild-type treated with BDNF, R6/2 mice and R6/2 mice treated with BDNF). The data obtained by image analysis were compared by means of one- way ANOVA including the group as main factor. Post hoc pair comparisons were carried out where necessary using HSD Tukey tests. P-values of less than 0.05 were considered to be statistically significant.

#### 3. Evaluation of NIIs

The NIIs were studied by means of single label immunofluorescence for ubiquitinated NIIs [57] using a mouse anti ubiquitin (Chemicon, Temecula, CA) with the previously described antigen retrieval method for immunohistochemistry [58].

A set of sections was counterstained with Nissl to calculate the number of neurons containing NIIs. The nucleus of each neuron was examined throughout its entire depth to ascertain whether an NII was present. A sample of about 250 neurons per hemisphere for each of three sections in each of 4 mice per treatment group was analyzed to determine the percentage of striatal neurons in R6/2 mice containing NIIs.

### Statistical Analysis

The data collected were analyzed to compare the effect of systemic BDNF on surviving cell number, size, NIIs size and percentage, BDNF expression and CREB and ERK activation in the striata of differently treated groups. Statistical analysis was performed by one or two-way ANOVA between or within groups including group or treatment as principal factors, followed by HSD Tukey test. P values of less than 0.05 were considered to be statistically significant.

### Ethics Statement

All studies were conducted in accordance with European Communities Council Directive of 24 November 1986 (86/609/EEC) and approved by the Santa Lucia Foundation Animal Care and Use committee.
