# Correction: Systemic Delivery of Recombinant Brain Derived Neurotrophic Factor (BDNF) in the R6/2 Mouse Model of Huntington's Disease

**DOI:** 10.1371/journal.pone.0166102

**Published:** 2016-11-23

**Authors:** Carmela Giampà, Elena Montagna, Clemente Dato, Mariarosa A. B. Melone, Giorgio Bernardi, Francesca Romana Fusco

There is information missing from the Funding section. The correct funding information is as follows: “The authors received financial support for the research and publication of this article by the Italian Department of Health grant number: GR -2010-2316475 (CG) and by grant RC12C. The funders had no role in study design, data collection and analysis, decision to publish, or preparation of the manuscript.”.
